# Identification of rare variants in candidate genes associated with monogenic diabetes in polish mody-x patients

**DOI:** 10.1007/s40200-023-01312-3

**Published:** 2023-10-13

**Authors:** Paulina Jakiel, K. Gadzalska, E. Juścińska, M. Gorządek, T. Płoszaj, S. Skoczylas, M. Borowiec, A. Zmysłowska

**Affiliations:** Department of Clinical Genetics, Medical University of Lodz, Lodz, Poland

**Keywords:** Candidate gene, MODY, MODY-X, Monogenic diabetes, Next-generation sequencing

## Abstract

**Purpose:**

Monogenic diabetes (MD) is caused by a mutation in a single gene and accounts for approximately 2.5–6% of all diabetes cases. Maturity-onset diabetes of the young (MODY) is the most common form of MD. To date, 14 different genes have been identified and associated with the presence of MODY phenotype. However, the number of potential candidate genes with relevance to beta cell function and glucose metabolism is increasing as more research is published. The aim of the study was to identify potentially causative variants in selected candidate genes in patients with a clinical diagnosis of MD.

**Methods:**

Targeted Next-Generation Sequencing (tNGS) on Illumina NextSeq 550 platform involving Agilent SureSelectQXT Target Enrichment protocol for 994 patients with suspected MD was performed. In the next step, the sequencing data of 617 patients with no pathogenic variants in main MD-related genes were reanalysed for the presence of causative variants in six candidate genes (*MTOR, TBC1D4, CACNA1E, MNX1, SLC19A2, KCNH6*). The presence of the selected variants was confirmed by Sanger sequencing.

**Results:**

Seven heterozygous possibly damaging variants were identified in four candidate genes (*MTOR, TBC1D4, CACNA1E, MNX1*). Five changes were assessed as novel variants, not previously described in available databases. None of the described variants were present among patients previously diagnosed with MODY diabetes due to causative, pathogenic variants in known MODY-related genes.

**Conclusions:**

The results obtained seem to confirm the effectiveness of the NGS method in identifying potentially causative variants in novel candidate genes associated with MODY diabetes.

## Introduction

Monogenic diabetes (MD), including maturity-onset diabetes of the young (MODY), neonatal diabetes mellitus (NDM) and syndromic forms of diabetes, are rare diseases caused by pathogenic/ likely pathogenic variants of a single gene. It is estimated that monogenic forms of diabetes represent around 2.5–6% of all diabetes cases [1, 2]. MODY diabetes is mainly diagnosed in adolescents or adults and is the most common type of MD [3–5]. Classically, MODY diabetes is characterised by mild/progressive hyperglycaemia, autosomal dominant inheritance, early onset of diabetes, absence of ketoacidosis and autoantibodies characteristic for autoimmune forms of diabetes, preserved insulin secretion, normal BMI and positive family history of diabetes [2, 6, 7].

To date, variants in at least 14 different genes have been identified as a cause of MODY diabetes. They include: *GCK, HNF1A, HNF4A, HNF1B, INS, NEUROD1, PDX1, PAX4, ABCC8, KCNJ11, KLF11, CEL, BLK* and *APPL1* genes. Pathogenic variants in the glucokinase gene (*GCK*), hepatocyte nuclear factor 1α (*HNF1A*) and hepatocyte nuclear factor 4 alpha (*HNF4A*) are most commonly found in patients with suspected MD [8].

In the latest large studies conducted among Polish patients with a suspected diagnosis of MD, the highest number of heterozygous causative variants was confirmed in the *GCK* and *HNF1A* genes [9, 10], which is consistent with the results obtained in the similar studies in France [11], Russia [12], Lithuania [13], North America [14] and Canada [15]. Another study conducted among 45 Polish patients with long-term T1DM without advanced complications, identified 9 patients as carriers of 10 variants in 4 genes associated with MODY diabetes (*ABCC8, GCK, HNF1A, HNF1B*), confirming that MODY cases are frequently misdiagnosed as type 1 or type 2 diabetes [16].

However, still a significant number of patients remain undiagnosed and genetic background of their disease is unknown (the so-called MODY-X cases). In individual cases, other genes may also be involved in the aetiology of MODY diabetes [17, 18]. Candidate genes are still identified based on their involvement in carbohydrate metabolism and insulin signalling [19].

Next-generation sequencing (NGS) is a powerful method, that may be used to discover novel causative genes in the MODY-X cases. Correct genetic diagnosis is important for determining the appropriate treatment for patients with diabetes [17, 19].

In the present study, NGS analysis was performed in a cohort of Polish patients with the MODY clinical phenotype to increase the possibility of identifying the pathogenic variant in the novel candidate genes that we selected: *MTOR, TBC1D4, CACNA1E, MNX1, SLC19A2, KCNH6*.

## Materials and methods

### Subjects

The study group consisted of 994 subjects (F/M; 48.9/ 51.1%), referred to the Genetic Outpatient Clinic of the Centre for Rare Diseases in Lodz, Poland (between 2016 and 2022) with suspected MD [10, 19]. The median age of the study participants at diagnosis was 12 years.

The inclusion clinical criteria for the study were as follows: presence of hyperglycaemia (H) or diabetes mellitus (DM) recognised according to the WHO definition, positive family history of diabetes, preserved insulin secretion and absence of autoantibodies and ketoacidosis at clinical diagnosis. The following parameters were analysed for all patients: HbA1c value (%) and fasting C-peptide value (ng/mL) at the time of clinical diagnosis of H/DM, the presence of T1DM-specific antibodies and body mass index (BMI). The clinical characteristics of the study participants are shown in Table 1.

**Table 1:** Clinical characteristics of study participants

Parameter	Total (n = 994)
Male, n (%)	51.1%
Female, n (%)	48.9%
Age at clinical diagnosis of H/DM (years)	12 (8–16)
Age at genetic testing (years)	14 (10–18)
HbA1c level at clinical diagnosis of H/DM (%)	6.1 (5.6–6.6)
C-peptide level at clinical diagnosis of H/DM (ng/mL)	1.31 (0.83-2.00)
BMI (kg/m^2)^	19.3 (16.5–22.2)

Data are shown as Me (IQR 25-75%) or n (%). Cases with data missing was excluded from analysis

H-hyperglycemia, DM-diabetes mellitus, BMI- body mass index, Me- median, IQR- interquartile range

The project was approved by the Bioethics Committee of the Medical University of Lodz (RNN/148/13/KE). Patients gave written informed consent for participation in the study.

### Molecular analysis

Genomic DNA was isolated from peripheral blood samples using an automated Maxwell system (Maxwell® RSC Blood DNA Kit, Promega, Madison, USA). The quantitative and qualitative assessment of the extracted DNA was performed on a NanoPhotometer® N80 (Implen, USA). The library was prepared using the Agilent SureSelectQXT Target Enrichment protocol with a custom gene panel according to the manufacturer’s instructions. Into the wells of a 96-well plate, 5 µl each of 10ng/µl DNA samples were transferred and subjected to enzymatic fragmentation. Then adapters were added to the ends of the fragments in a single reaction. The libraries were purified using AMPure XP magnetic beads. Then, the adaptor-tagged gDNA libraries were repaired and PCR-amplified. The products were again purified using AMPure XP magnetic beads. The concentration and size of the resulting product were assessed using an Agilent TapeStation and a dedicated D1000 ScreenTape and reagent kit. In the next step, the prepared gDNA samples were hybridised to the target-specific Capture Library. Then the hybridised DNA was captured using streptavidin-coated beads and enriched DNA libraries were PCR- amplified using appropriate pair of dual indexing primers. The resulting products were again purified using AMPure XP magnetic beads. The concentrations and sizes of the indexed DNA libraries were assessed using an Agilent TapeStation and a dedicated HS1000 ScreenTape and reagent kit. The indexed samples were pooled, diluted and combined with PhiX control. Targeted next generation sequencing (NGS) was performed on Illumina NextSeq 550 platform, (2 × 150 bp).

Initially, a 15-gene NGS panel for MD including *HNF4A, GCK, HNF1A, PAX4, HNF1B, NEUROD1, APPL1, KLF11, CEL, BLK, PDX1, ABCC8, KCNJ11, INS* and *WFS1* was analysed. In the next step, the sequencing data of patients with no pathogenic variants in the 15 studied genes were reanalysed for the presence of causative variants in six candidate genes: *MTOR, TBC1D4, CACNA1E, MNX1, SLC19A2, KCNH6*. The candidate genes were selected based on their involvement in carbohydrate metabolism and insulin signalling [20–25].

### Bioinformatic analysis

Alignments and variant calling were performed using BWA Enrichment v2.1 BaseSpace application (Illumina, San Diego, USA). Variant annotations and filtering were carried out in Illumina Variant Studio Software (Illumina, San Diego, USA). Minor allele frequency (MAF) < 0.01 for the European population in the GnomAD and ExAC database was used to filter significant variants in the candidate genes. The potential damaging role of the variants was determined *in silico* using web-based software, such as PROVEAN and Mutation Taster. The pathogenicity of detected genetic variants was evaluated according to the guidelines of the American College of Medical Genetics and Genomics (ACMG) [26].

### Variant confirmation

The presence of selected variants was confirmed by bidirectional Sanger sequencing on a 3500 Series Genetic Analyzer (Applied Biosystems, Waltham, MA, USA). Sequence analysis was performed using Sequencher 5.0 software.

### Statistical analysis

The normality of linear values distribution was assessed using the Shapiro-Wilk test. Medians (Me) and interquartile ranges (IQR 25-75%) were used to present continuous variables. Nominal values were presented as a percentage (%) for the study group. Statistical analysis was performed using STATISTICA 13.3 software (Statsoft, Tulsa, OK, USA).

## Results

**Fig. 1:**
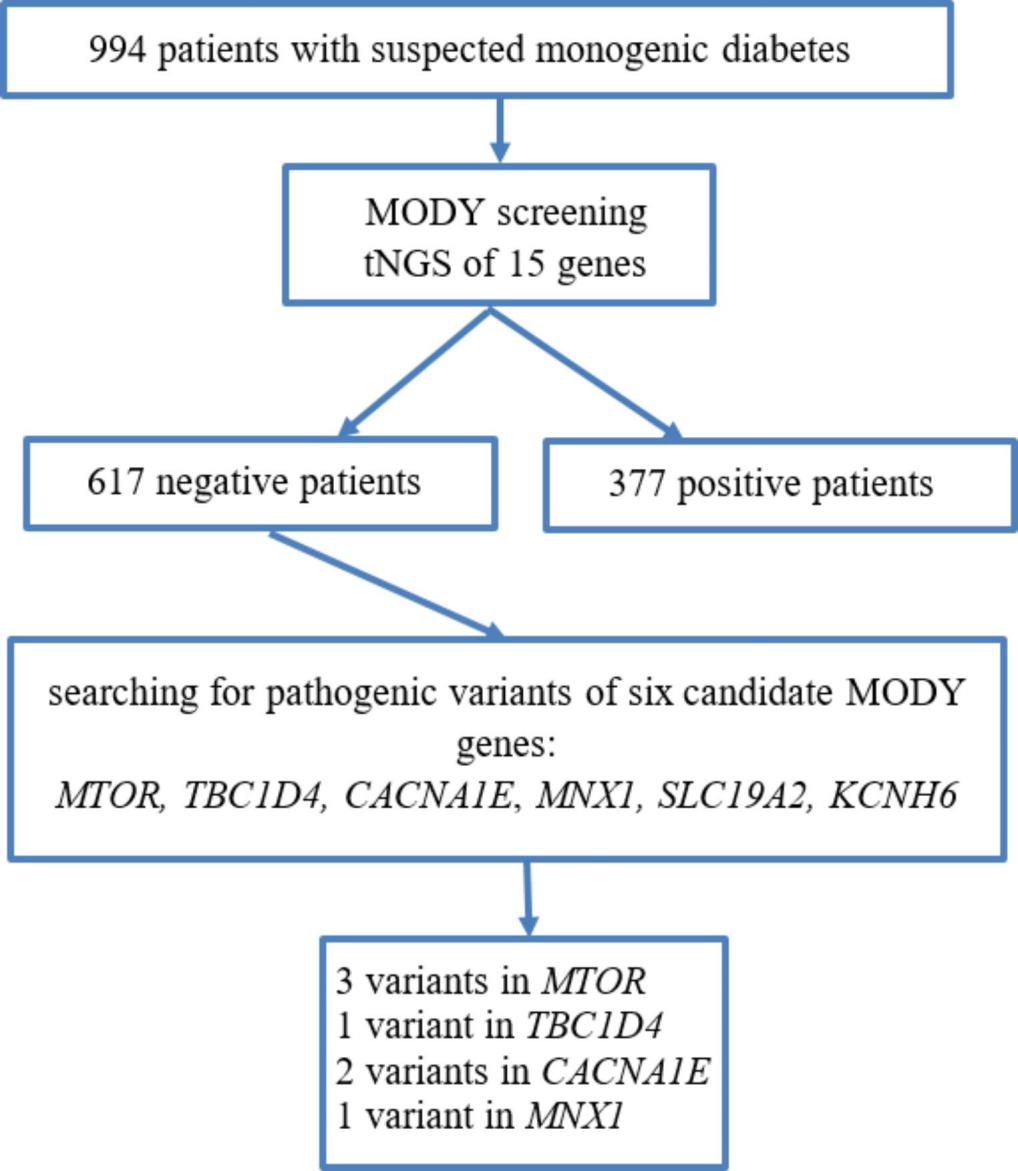
Details of molecular diagnosis in patients with suspected monogenic diabetes

A total of 994 patients with suspected MD were included in the study. Pathogenic and likely pathogenic variants responsible for causing MD were identified in 377 patients and were found in eight different MD-related genes: *ABCC8, GCK, HNF1A, HNF1B, HNF4A, KCNJ11, PDX1, WFS1*. Subsequently, reanalysis of the sequencing data revealed seven heterozygous possibly causative candidate gene variants in the negative patient cohort. Three of them were in the *MTOR* gene, one in the *TBC1D4*, two in the *CACNA1E* and one in the *MNX1* gene. No pathogenic or likely pathogenic variant was identified in the *SLC19A2* and *KCNH6* genes that could potentially cause MODY diabetes in the study group (Fig. 1).

Details of the identified variants are contained in Table 2. Among the seven variants identified in the candidate genes, four were missense, one frameshift, one in- frame deletion and one start-lost mutations. Five changes were assessed as novel variants not previously described in available databases. Moreover, high conservativeness in the studied variants (except p.His196_Ala198del in *MNX1* gene) was confirmed among five species (*G.gorilla, M.musculus, C. lupus, O. cuniculus, S. scrofa)*. The selected variants were identified in a total of seven patients. The clinical characteristics of these patients are presented in Table 3.

**Table 2:** Genetic characteristics of MODY candidate variants

Patient ID	Chromosome coordinates	Gene	HGVSc	HGVSp	dbSNP	ClinVar	gnomAD EUR (%)	Mutation Taster prediction (score)	PROVEAN Prediction (score)	SIFT Prediction (score)	ACMG classification
#1	chr1:11187709	*MTOR*	NM_004958.3:c.6187delC	NP_004949.1:p.Gln2063ArgfsTer3							Likely pathogenic
#2	chr1:11291014	*MTOR*	NM_004958.3:c.2747G > A	NP_004949.1:p.Ser916Asn				Disease causing (1)	Neutral (-1.99)	Tolerated (0.419)	VUS
#3	chr1:11291394	*MTOR*	NM_004958.3:c.2612T > G	NP_004949.1:p.Phe871Cys				Disease causing (1)	Damaging (-6.26)	Damaging (0,005)	VUS
#4	chr13:76055902	*TBC1D4*	NM_014832.2:c.2T > C	NP_055647.2:p.Met1?	rs769998822			Disease causing (1)	Neutral (-0.4)	Damaging (0)	Likely pathogenic
#5	chr1:181727158	*CACNA1E*	NM_001205293.1:c.4405T > C	NP_001192222.1:p.Tyr1469His				Disease causing (1)	Damaging (-4.78)	Damaging (0)	VUS
#6	chr1:181620493	*CACNA1E*	NM_001205293.1:c.971 C > A	NP_001192222.1:p.Ala324Asp				Disease causing (0.9968)	Neutral (-0.34)	Tolerated (0.074)	VUS
#7	chr7:156802451	*MNX1*	NM_005515.3:c.585_593delGCACCCCGC	NP_005506.3:p.His196_Ala198del	rs528990697	VUS	ƒ = 0.0000208				VUS

**VUS- v**ariants of uncertain significance, **HGVSc**- nucleotide change described according to the HGVS (Human Genome Variation Society) nomenclature standard, **HGVSp-** protein change described according to the HGVS (Human Genome Variation Society) nomenclature standard, **gnomAD EUR (%)-**frequency in the gnomAD database for the European population

MutationTaster score – Scores ranges from 0 to 1. The larger the score, the more likely the variant is pathogenic

PROVEAN score - Scores range from − 14 to 14. The smaller the score the more likely the SNP has damaging effect

SIFT score - Scores range from 0 to 1. The smaller the score the more likely the SNP has damaging effect

**Table 3:** Clinical characteristics of the patients with selected variants of candidate genes

Patient ID	Gender (F/M)	H (IFG) /Diabetes	Age at study time (years)	Age at hyperglycemia/diabetes onset (years)	HbA1c level at clinical diagnosis of H/DM (%)	C-peptide level at clinical diagnosis of H/DM 0‘(ng/ml)	BMI (kg/m^2^)	Treatment	Hyperglycemia (H)/diabetes mellitus (DM) in a family
#1	F	Diabetes	14	13	5.4	2.06	14.3	Diet	Father, grandfather and grandmother - DM
#2	F	Diabetes	24	24	NA	NA	NA	Diet	No
#3	M	IFG	10	8	5.9	0.72	14.3	Diet	Father-H, grandfather-DM
#4	M	Diabetes	12	8	5.7	1.1	21.2	Insulin	No
#5	F	IFG	33	31	5.5	1.83	24	Diet	Mother and mom’s 3 sisters- DM
#6	M	Diabetes	1	6 months	7.7	0.7	13.8	Insulin	Father, grandfather-H
#7	M	Diabetes	58	53	6.9	1.21	23.8	Insulin	Siblings-DM

**H-**hyperglycemia; **IFG** – impaired fasting glycemia

The median age of clinical diagnosis of hyperglycaemia or diabetes in these patients was 13 years and was 1 year lower than the median age at the time of genetic testing (Me = 14.0 years). Five out of the seven patients (71%) had a positive family history of diabetes. The HbA1c level at clinical diagnosis was different among patients (IQR 25-75% − 5.5–6.9%). A decreased C-peptide level − 0.7 ng/ml (normal range: 0.9-4.0 ng/ml) was observed in two cases. Three patients were treated with insulin therapy, while four were exclusively on a diet with low glycaemic index (IG) at the time of the genetic diagnosis.

None of the described variants were present among 377 patients who were previously diagnosed with MODY diabetes due to changes in known MODY-related genes, which confirms that the selected changes are not the methodological errors or artifacts. In addition, the presence of selected variants was confirmed by bidirectional Sanger sequencing in all patients.

**Fig. 2:**
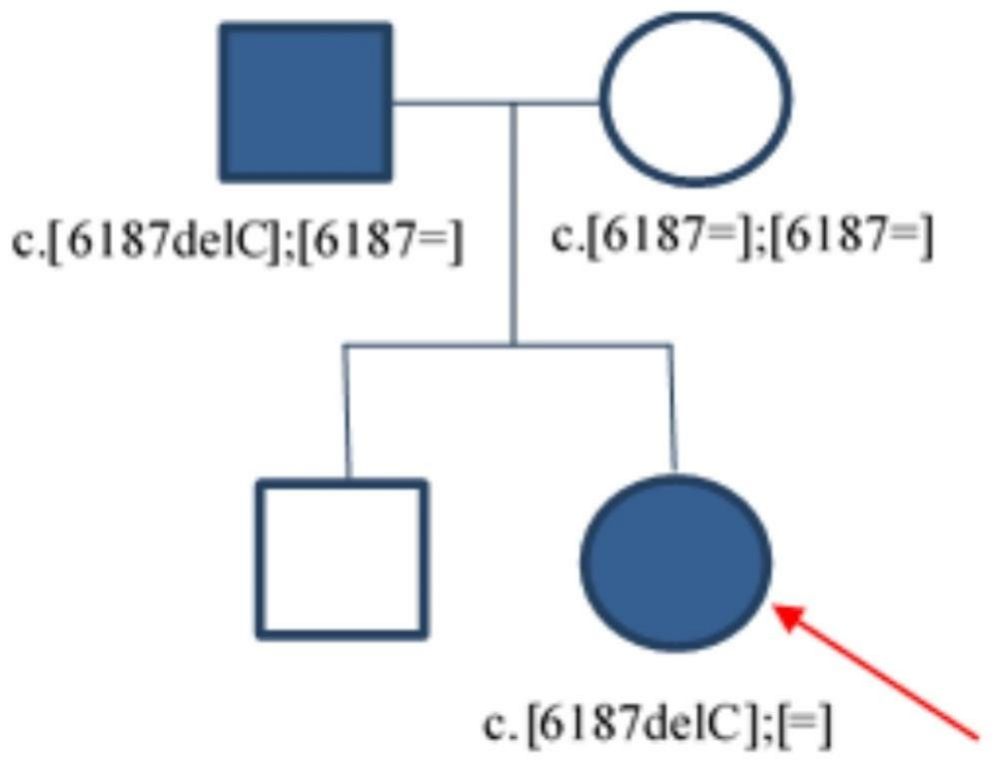
Pedigree of family with heterozygous c.6187delC variant in the *MTOR* gene. Genotype is shown underneath each symbol. Squares represent male family members, and circles represent female sex. Blue-filled symbols denote patients with diabetes, an arrow denotes the proband in the family

**Fig. 3:**
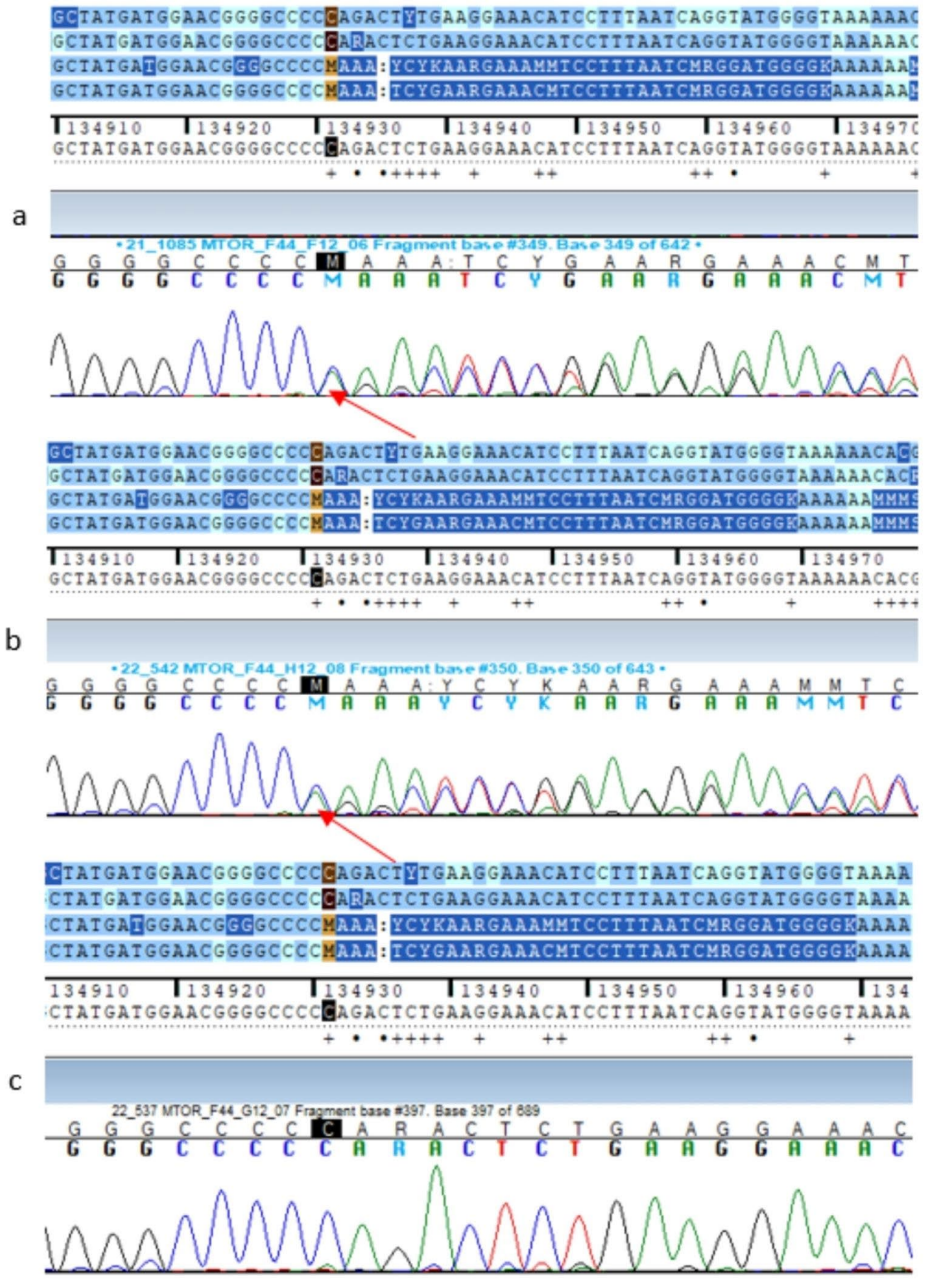
Sanger sequencing chromatogram showing the heterozygous c.6187delC variant in exon 44 of the *MTOR* gene identified in the proband and in her father (a-proband, b-father, c-mother)

The analysis of segregation in the family was only possible for the c.6187delC variant in the *MTOR* gene. A heterozygous c.6187delC variant was identified in a 14-year-old female patient with diabetes diagnosed at the age of 13 and in her father with suspected type 2 diabetes, while it was not present in her asymptomatic mother (Figs. 2 and 3).

## Discussion

This is the first study to analyse the sequence of the *MTOR, TBC1D4, CACNA1E, SLC19A2* and *KCNH6* genes in Polish patients with suspected MODY-X diabetes using the NGS method. Seven heterozygous possibly causative variants in candidate genes were identified in 7 of 617 previously undiagnosed patients with a positive family history, preserved insulin secretion and absence of autoantibodies characteristic for autoimmune diabetes. No potentially pathogenic variant associated with diabetes was detected in the remaining patients. According to the results, it is difficult to identify a specific type of monogenic diabetes in the above patients. Therefore, in such cases, whole exome sequencing (WES) could be an important complement to the diagnostics [17, 27].

In our study group, three of the selected variants are present in the *MTOR* gene encoding mammalian target of rapamycin (mTOR), which play an important role in regulating cell growth as well as lipid and glucose metabolism. Numerous studies have shown that *MTOR* is an important gene in the insulin signalling pathway and susceptibility to type 2 diabetes mellitus [20, 28, 29]. mTOR exists in two structurally and functionally distinct multiprotein complexes – mTORC1 and mTORC2. The effects of mTOR on glucose homeostasis are complex and depend on the level of mTORC1 activity. Activation of mTORC1 in β-cells increases insulin secretion and thus reduces blood glucose levels. However, a sustained activation of mTORC1 leads to exhaustion of insulin secreting capacity by β-cells and to impaired glucose homeostasis. There are several reports that treatment with mTOR inhibitors in various cancers is associated with a high incidence of hyperglycaemia and new-onset diabetes [30]. These findings could lead to the hypothesis that both gain-of-function and loss-of-function *MTOR* variants may cause hyperglycaemia or diabetes. All changes indicated in *MTOR* gene in this study are novel variants, not previously described in available databases (dbSNP, ClinVar, HGMD). There are no data on the frequency of these variants in the gnomAD and ExAC databases. Unfortunately, detailed clinical characteristics of the patient with the identified p.Ser916Asn variant were not available. However, the other two patients with the p.Gln2063ArgfsTer3 and p.Phe871Cys variants in the *MTOR* gene had a positive family history, normal C-peptide and HbA1c level, absence of autoantibodies characteristic for autoimmune diabetes and were exclusively on a nutrition therapy. The examination of the proband’s family confirmed the co-segregation of the c.6187delC (p.Gln2063ArgfsTer3) variant with diabetes, which may confirm variant pathogenicity. Based on the above, we could suppose that the *MTOR* gene is associated with rare cases of MODY-X.

Another variant was identified in the *TBC1D4* gene encoding the TBC1 domain family member 4 protein. This protein play an important role in glucose homeostasis by regulating trafficking of the glucose transporter 4 (GLUT4), which is insulin-dependent and important for removing glucose from the bloodstream into skeletal muscle and fat tissues [31]. The heterozygous start-loss variant c.2T > C (p.Met1?) identified in our patient has already been reported in the dbSNP database and is classified as likely pathogenic according to the ACMG guidelines. *In silico* analysis showed that this variant leads to the loss of the first methionine, thus precluding the translation initiation of the corresponding protein. Other potentially damaging variants of *TBC1D4* were previously reported to cause a higher risk of type 2 diabetes and insulin resistance [31, 32]. Moltke et al. identified a common nonsense variant (p.R684Ter) in the Greenlandic population in *TBC1D4*, which in homozygous carriers causes insulin resistance and increases the risk of type 2 diabetes. However, heterozygous carriers had only slightly higher plasma glucose concentration 2 h after an oral glucose load [32]. We suppose that rare heterozygous variants with more deleterious effects could be involved in MODY, while those with a smaller effect might have a role in type 2 diabetes. Similarly, variants of the *KCNJ11* and *HNF4A* genes could cause MODY or type 2 diabetes, depending on how the variant affects the protein function [33]. However, this is only a hypothesis and we cannot confirm that the variant in *TBC1D4* was directly causative and correlated with diabetes in our patient.

Next, two novel variants in the *CACNA1E* gene were identified. The nonsynonymous p.Tyr1469His variant is located in the 31st exon of the *CACNA1E* gene. Both predictive programs used indicate the damaging nature of this change. The second variant (p.Ala324Asp) is located in the seventh exon. Predictive programs show conflicting information about the pathogenicity of this variant. Both variants were classified as variants of uncertain significance (VUS) according to the ACMG guidelines. C*ACNA1E* was selected as a candidate gene because of their previously reported role in the risk of type 2 diabetes, insulin resistance, impaired insulin secretion and regulation of β-cell function [22, 34]. The generation of β cell electrical activity that results from the functional interaction of the ATP-sensitive beta cell K + channel (encoded by the *KCNJ11* gene) and voltage-dependent Ca2 + channels (VDCCs) is required to insulin secretion. Human pancreatic β cells express several types of VDCCs including the CaV1.2 and CaV2.3. The CaV2.3 channel, encoded by *CACNA1E* gene is responsible for sustained second-phase insulin release. The absence of the CaV2.3 channel in mice causes fasting hyperglycaemia and reduced glucose clearance. Holkmvist et al. concluded that the Ca_V_2.3 channel could play an important role in regulating insulin secretion, thus *CACNA1E* is an relevant candidate gene in type 2 diabetes [35]. The presence of two potentially causative *CACNA1E* variants in two patients from our cohort seems to suggest that this gene may also play a role in monogenic diabetes. In addition, patients with the p.Ala324Asp variant had slightly decreased C-peptide levels (0.7 ng/ml), which could indicate a defect in insulin secretion caused by a change in the *CACNA1E* gene. Thus, by analogy, it can be concluded that rare severe variants in the *CACNA1E* gene may cause monogenic diabetes, as is the case with the *HNF1β, GCK* or *KCNJ11* genes, while common single nucleotide alterations are associated with an increased risk of type 2 diabetes [36].

The last variant was identified in the first exon of the *MNX1* gene which encodes a homeobox transcription factor described as important for pancreatic beta cell differentiation and development [23, 37]. The p.His196_Ala198del variant identified in the present study has been previously reported in the dbSNP and ClinVar databases as VUS. The prevalence of this variant in the gnomAD database is 0.0000208 for the European population. Therefore, it can be concluded that this is a very rare variant, classified as VUS according to the ACMG criteria. Heterozygous loss of function variants in *MNX1* cause Currarino syndrome, a rare clinical condition that is characterised by presacral mass, sacral agenesis and an anorectal anomaly [23]. Several homozygous variants in the *MNX1* gene have already been described in the literature as associated with the phenotype of persistent neonatal diabetes mellitus (PNDM). All of these patients were diagnosed in infancy [23, 37, 38]. In contrast, a heterozygous variant was identified in our patient, who was diagnosed with diabetes at the age of 53. It is possible that, similarly to the *GCK* and *RFX6* genes, biallelic variants cause a NDM, while the heterozygous variants are associated with MODY diabetes [3, 39].

Unfortunately, our study has several limitations. Due to the lack of available DNA from relatives, it was not possible to perform a family segregation analysis for all variants, and the inference is based only on *in silico* prediction and descriptions in available databases. Similarly, we did not have the BMI values, C-peptide level and HbA1c level for one patient with the *MTOR* gene variant, which could have enriched the clinical characteristics of patients with the *MTOR* gene changes. Another limitation of the present study is that no functional studies have been conducted in order to explore the biological relevance of the variants on glucose metabolism. Furthermore, the final confirmation of the effect of selected candidate genes on the MODY disease phenotype requires testing on a larger group of carriers, performing a family segregation analysis and functional studies.

In conclusion, the results obtained in this study seem to confirm the effectiveness of the NGS method in identifying potentially pathogenic variants in novel candidate gene related to carbohydrate metabolism and insulin signalling.

The use of multigene panel testing based on next-generation sequencing increases the chance of accurately diagnosing extremely rare forms of MODY, and thus implementing appropriate treatment in patients. However, still a significant number of patients remain undiagnosed, and in such cases, WES or WGS methods can play a key role in understanding the genetic basis of the disease. In the future, it is planned to conduct further studies on a larger group of patients with suspected MD, obtain biological material from family members and conduct a family segregation analysis to definitively confirm or exclude the influence of selected candidate genes on the MODY disease phenotype.
